# Supplementary material for: Artificially stimulating retrotransposon activity increases mortality and accelerates a subset of aging phenotypes in Drosophila
Source: eLife. 2022 Aug 18;11:e80169. doi: 10.7554/eLife.80169 (PMC9427105; doi:10.7554/eLife.80169)
Supplement: Supplementary file 1. [file elife-80169-supp1.docx]

| TE | wt_6d_1 | wt_6d_2 | wt_5d_3 | wt_30d_1 | wt_30d_2 | wt_30d_3 | adjusted p value | Fold Change | Class |
| --- | --- | --- | --- | --- | --- | --- | --- | --- | --- |
| *17.6* | 57 | 108 | 49 | 29 | 100 | 98 | 9.97E-01 | 1.06 | Retrotransposon |
| *297* | 722 | 1071 | 787 | 68 | 409 | 684 | 9.39E-01 | 0.45 | Retrotransposon |
| *412* | 136 | 174 | 95 | 61 | 126 | 238 | 9.97E-01 | 1.05 | Retrotransposon |
| *1731* | 1432 | 2236 | 1051 | 3987 | 6466 | 7530 | 2.10E-04 | 3.81 | Retrotransposon |
| *ACCORD* | 49 | 436 | 21 | 1 | 40 | 157 | 9.97E-01 | 0.39 | Retrotransposon |
| *aurora-element* | 41 | 27 | 27 | 35 | 29 | 28 | 9.97E-01 | 0.98 | Retrotransposon |
| *BAGGINS* | 28 | 43 | 19 | 12 | 31 | 60 | 9.97E-01 | 1.14 | Retrotransposon |
| *Beagle* | 486 | 391 | 455 | 343 | 400 | 369 | 9.97E-01 | 0.83 | Retrotransposon |
| *Beagle2* | 23 | 56 | 11 | 11 | 25 | 54 | 9.97E-01 | 0.99 | Retrotransposon |
| *Bel* | 1000 | 1365 | 314 | 254 | 882 | 1594 | 9.97E-01 | 1.02 | Retrotransposon |
| *BLOOD* | 488 | 578 | 505 | 135 | 499 | 381 | 9.97E-01 | 0.65 | Retrotransposon |
| *BS* | 63 | 84 | 27 | 26 | 49 | 66 | 9.97E-01 | 0.81 | Retrotransposon |
| *BS3* | 66 | 60 | 23 | 65 | 59 | 52 | 9.97E-01 | 1.17 | Retrotransposon |
| *Burdock* | 1044 | 1138 | 704 | 900 | 1811 | 1675 | 9.96E-01 | 1.52 | Retrotransposon |
| *CIRC* | 43 | 41 | 17 | 14 | 45 | 73 | 9.97E-01 | 1.30 | Retrotransposon |
| *COPIA* | 78001 | 138845 | 60568 | 95444 | 238381 | 250318 | 6.22E-01 | 2.11 | Retrotransposon |
| *CR1A* | 333 | 463 | 124 | 142 | 344 | 419 | 9.97E-01 | 0.98 | Retrotransposon |
| *Diver* | 131 | 127 | 19 | 7 | 71 | 121 | 9.97E-01 | 0.72 | Retrotransposon |
| *DIVER2* | 62 | 51 | 10 | 4 | 33 | 46 | 9.97E-01 | 0.68 | Retrotransposon |
| *DM_ROO* | 1320 | 1461 | 521 | 898 | 1373 | 1649 | 9.97E-01 | 1.19 | Retrotransposon |
| *DM88* | 57 | 62 | 16 | 22 | 50 | 66 | 9.97E-01 | 1.02 | Retrotransposon |
| *DMGYPF1A* | 777 | 984 | 454 | 311 | 644 | 862 | 9.97E-01 | 0.82 | Retrotransposon |
| *DOC* | 2501 | 4171 | 1162 | 480 | 2344 | 3052 | 9.97E-01 | 0.75 | Retrotransposon |
| *DOC2* | 241 | 303 | 40 | 52 | 151 | 248 | 9.97E-01 | 0.77 | Retrotransposon |
| *DOC3* | 114 | 134 | 63 | 84 | 146 | 138 | 9.97E-01 | 1.18 | Retrotransposon |
| *DOC4* | 105 | 177 | 88 | 525 | 422 | 359 | 1.00E-05 | 3.52 | Retrotransposon |
| *DOC5* | 8 | 18 | 9 | 14 | 26 | 36 | 1.00E+00 | 2.14 | Retrotransposon |
| *F-element* | 392 | 646 | 138 | 74 | 304 | 594 | 9.97E-01 | 0.83 | Retrotransposon |
| *Flea* | 1122 | 1506 | 397 | 310 | 1042 | 1294 | 9.97E-01 | 0.87 | Retrotransposon |
| *FROGGER* | 59 | 62 | 6 | 7 | 42 | 57 | 9.97E-01 | 0.84 | Retrotransposon |
| *FW2* | 38 | 44 | 15 | 19 | 45 | 41 | 9.97E-01 | 1.08 | Retrotransposon |
| *FW3* | 43 | 38 | 27 | 34 | 36 | 42 | 9.97E-01 | 1.04 | Retrotransposon |
| *G2* | 87 | 71 | 33 | 28 | 56 | 73 | 9.97E-01 | 0.82 | Retrotransposon |
| *G3* | 15 | 21 | 12 | 4 | 22 | 23 | 1.00E+00 | 1.02 | Retrotransposon |
| *G4* | 129 | 142 | 74 | 73 | 130 | 160 | 9.97E-01 | 1.05 | Retrotransposon |
| *G5* | 57 | 74 | 11 | 27 | 70 | 72 | 9.97E-01 | 1.19 | Retrotransposon |
| *G5A* | 184 | 185 | 155 | 147 | 175 | 218 | 9.97E-01 | 1.03 | Retrotransposon |
| *G6* | 122 | 237 | 253 | 22 | 79 | 59 | 8.30E-03 | 0.26 | Retrotransposon |
| *Gate* | 252 | 288 | 69 | 240 | 563 | 881 | 3.95E-01 | 2.76 | Retrotransposon |
| *GTWIN* | 28 | 78 | 13 | 36 | 70 | 62 | 9.97E-01 | 1.40 | Retrotransposon |
| *GYPSY10* | 179 | 225 | 102 | 110 | 197 | 228 | 9.97E-01 | 1.06 | Retrotransposon |
| *GYPSY11* | 24 | 20 | 4 | 12 | 11 | 15 | 1.00E+00 | 0.80 | Retrotransposon |
| *GYPSY2* | 29 | 33 | 13 | 23 | 36 | 38 | 9.97E-01 | 1.26 | Retrotransposon |
| *GYPSY4* | 52 | 91 | 33 | 35 | 68 | 81 | 9.97E-01 | 1.05 | Retrotransposon |
| *GYPSY5* | 22 | 33 | 10 | 10 | 38 | 21 | 1.00E+00 | 1.05 | Retrotransposon |
| *GYPSY6* | 25 | 7 | 13 | 17 | 18 | 13 | 1.00E+00 | 1.04 | Retrotransposon |
| *GYPSY8* | 53 | 29 | 17 | 25 | 54 | 70 | 9.97E-01 | 1.50 | Retrotransposon |
| *GYPSY9* | 59 | 56 | 26 | 33 | 52 | 43 | 9.97E-01 | 0.91 | Retrotransposon |
| *HeT-A* | 1110 | 1252 | 658 | 729 | 1253 | 1494 | 9.97E-01 | 1.15 | Retrotransposon |
| *I-element* | 270 | 552 | 105 | 85 | 349 | 509 | 9.97E-01 | 1.02 | Retrotransposon |
| *Idefix* | 141 | 250 | 86 | 105 | 239 | 309 | 9.97E-01 | 1.36 | Retrotransposon |
| *INVADER* | 49 | 63 | 18 | 31 | 53 | 66 | 9.97E-01 | 1.15 | Retrotransposon |
| *INVADER2* | 47 | 145 | 19 | 22 | 103 | 158 | 9.97E-01 | 1.34 | Retrotransposon |
| *INVADER3* | 70 | 123 | 67 | 140 | 127 | 128 | 9.23E-01 | 1.51 | Retrotransposon |
| *INVADER4* | 36 | 29 | 10 | 35 | 47 | 73 | 8.52E-01 | 2.07 | Retrotransposon |
| *IVK* | 95 | 167 | 16 | 18 | 156 | 218 | 9.97E-01 | 1.40 | Retrotransposon |
| *Jockey* | 644 | 705 | 330 | 316 | 556 | 769 | 9.97E-01 | 0.98 | Retrotransposon |
| *Jockey_1* | 282 | 386 | 212 | 263 | 334 | 405 | 9.97E-01 | 1.14 | Retrotransposon |
| *JOCKEY2* | 219 | 196 | 108 | 201 | 199 | 166 | 9.97E-01 | 1.08 | Retrotransposon |
| *JUAN* | 87 | 82 | 23 | 17 | 73 | 77 | 9.97E-01 | 0.87 | Retrotransposon |
| *Max-element* | 2543 | 2091 | 1407 | 2719 | 2744 | 3012 | 9.96E-01 | 1.40 | Retrotransposon |
| *MDG3* | 288 | 418 | 104 | 143 | 437 | 505 | 9.97E-01 | 1.34 | Retrotransposon |
| *MGD1* | 298 | 346 | 134 | 214 | 382 | 580 | 9.97E-01 | 1.51 | Retrotransposon |
| *Microcopia* | 19 | 19 | 5 | 11 | 25 | 30 | 1.00E+00 | 1.52 | Retrotransposon |
| *ninja* | 43 | 39 | 26 | 44 | 52 | 67 | 9.97E-01 | 1.52 | Retrotransposon |
| *OPUS* | 1991 | 2046 | 588 | 1730 | 3516 | 4090 | 8.73E-01 | 2.02 | Retrotransposon |
| *QBERT* | 46 | 51 | 19 | 43 | 73 | 82 | 9.97E-01 | 1.68 | Retrotransposon |
| *QUASIMODO* | 47 | 53 | 17 | 17 | 39 | 62 | 9.97E-01 | 1.00 | Retrotransposon |
| *R1-2* | 21 | 23 | 11 | 12 | 11 | 17 | 1.00E+00 | 0.73 | Retrotransposon |
| *R1A1-element* | 7 | 27 | 11 | 28 | 26 | 25 | 1.00E+00 | 1.73 | Retrotransposon |
| *ROOA_LTR* | 382 | 444 | 69 | 144 | 454 | 1048 | 9.97E-01 | 1.84 | Retrotransposon |
| *ROVER* | 64 | 133 | 106 | 127 | 289 | 348 | 1.44E-01 | 2.52 | Retrotransposon |
| *Rt1a* | 32 | 54 | 50 | 11 | 43 | 36 | 9.97E-01 | 0.67 | Retrotransposon |
| *RT1B* | 179 | 193 | 47 | 110 | 227 | 167 | 9.97E-01 | 1.20 | Retrotransposon |
| *RT1C* | 24 | 27 | 7 | 17 | 27 | 20 | 1.00E+00 | 1.07 | Retrotransposon |
| *S-element* | 102 | 134 | 37 | 69 | 97 | 136 | 9.97E-01 | 1.10 | Retrotransposon |
| *S2* | 21 | 29 | 17 | 25 | 23 | 33 | 1.00E+00 | 1.20 | Retrotransposon |
| *SPRINGER* | 723 | 876 | 409 | 655 | 1035 | 1186 | 9.97E-01 | 1.43 | Retrotransposon |
| *STALKER2* | 450 | 745 | 348 | 130 | 178 | 214 | 1.07E-03 | 0.34 | Retrotransposon |
| *TABOR* | 149 | 240 | 54 | 71 | 169 | 258 | 9.97E-01 | 1.12 | Retrotransposon |
| *TARTC* | 106 | 189 | 51 | 34 | 104 | 141 | 9.97E-01 | 0.81 | Retrotransposon |
| *TOM1_LTR* | 72 | 75 | 91 | 110 | 93 | 89 | 9.97E-01 | 1.23 | Retrotransposon |
| *Transpac* | 569 | 833 | 296 | 273 | 953 | 973 | 9.97E-01 | 1.29 | Retrotransposon |
| *X-ELEMENT* | 67 | 115 | 21 | 15 | 58 | 102 | 9.97E-01 | 0.86 | Retrotransposon |
| *ZAM* | 24 | 41 | 29 | 7 | 34 | 28 | 9.97E-01 | 0.73 | Retrotransposon |
| *1360* | 1126 | 1263 | 543 | 916 | 1226 | 1333 | 9.97E-01 | 1.18 | DNA |
| *BARI1* | 82 | 78 | 45 | 64 | 99 | 77 | 9.97E-01 | 1.16 | DNA |
| *Hobo* | 365 | 463 | 237 | 139 | 384 | 390 | 9.97E-01 | 0.86 | DNA |
| *Pogo* | 720 | 678 | 801 | 792 | 889 | 741 | 9.97E-01 | 1.10 | DNA |
| *HB* | 161 | 215 | 40 | 53 | 167 | 173 | 9.97E-01 | 0.94 | DNA |
| *FB* | 200 | 185 | 146 | 197 | 237 | 244 | 9.97E-01 | 1.27 | DNA |
| *Hopper* | 24 | 21 | 19 | 16 | 31 | 22 | 1.00E+00 | 1.05 | DNA |
| *INE1* | 164 | 208 | 96 | 104 | 196 | 207 | 9.97E-01 | 1.08 | DNA |
| *MARINER2* | 97 | 78 | 69 | 104 | 103 | 96 | 9.97E-01 | 1.24 | DNA |
| *P-element* | 432 | 405 | 285 | 322 | 421 | 314 | 9.97E-01 | 0.94 | DNA |
| *TC1* | 126 | 156 | 29 | 43 | 90 | 147 | 9.97E-01 | 0.89 | DNA |
| *TC1-2* | 59 | 133 | 55 | 44 | 103 | 161 | 9.97E-01 | 1.24 | DNA |
| *TRANSIB2* | 123 | 102 | 49 | 71 | 115 | 117 | 9.97E-01 | 1.10 | DNA |
| *TRANSIB3* | 90 | 166 | 44 | 136 | 205 | 231 | 8.73E-01 | 1.90 | DNA |
| *TRANSIB4* | 7 | 20 | 9 | 7 | 11 | 20 | 1.00E+00 | 1.07 | DNA |
