# Supplementary material for: Artificially stimulating retrotransposon activity increases mortality and accelerates a subset of aging phenotypes in Drosophila
Source: eLife. 2022 Aug 18;11:e80169. doi: 10.7554/eLife.80169 (PMC9427105; doi:10.7554/eLife.80169)
Supplement: Supplementary file 2. [file elife-80169-supp2.docx]

| TE | Δ94_6d_1 | Δ94_6d_2 | Δ94_6d_3 | Δ94_31d_1 | Δ94_31d_2 | Δ94_30d_3 | adjusted p value | Fold Change | Class |
| --- | --- | --- | --- | --- | --- | --- | --- | --- | --- |
| *17.6* | 28 | 67 | 58 | 81 | 90 | 73 | 1.50E-01 | 1.60 | Retrotransposon |
| *297* | 194 | 506 | 164 | 949 | 1081 | 270 | 1.78E-01 | 2.66 | Retrotransposon |
| *412* | 125 | 256 | 223 | 284 | 129 | 58 | 6.18E-01 | 0.78 | Retrotransposon |
| *1731* | 354 | 427 | 645 | 834 | 1605 | 2198 | 5.00E-05 | 3.25 | Retrotransposon |
| *ACCORD* | 1077 | 1259 | 1875 | 2598 | 4876 | 1519 | 2.47E-02 | 2.14 | Retrotransposon |
| *aurora-element* | 19 | 16 | 22 | 9 | 13 | 22 | 5.68E-01 | 0.74 | Retrotransposon |
| *BAGGINS* | 33 | 33 | 29 | 47 | 40 | 34 | 4.82E-01 | 1.29 | Retrotransposon |
| *Beagle* | 112 | 211 | 270 | 265 | 328 | 248 | 2.41E-01 | 1.42 | Retrotransposon |
| *Beagle2* | 8 | 21 | 25 | 29 | 53 | 22 | 1.51E-01 | 1.98 | Retrotransposon |
| *Bel* | 873 | 732 | 620 | 1128 | 894 | 1026 | 1.03E-01 | 1.37 | Retrotransposon |
| *BLOOD* | 718 | 1574 | 1252 | 1974 | 1922 | 1453 | 1.33E-01 | 1.51 | Retrotransposon |
| *BS* | 272 | 288 | 307 | 395 | 355 | 536 | 4.52E-02 | 1.48 | Retrotransposon |
| *BS3* | 47 | 46 | 54 | 41 | 38 | 41 | 5.29E-01 | 0.81 | Retrotransposon |
| *Burdock* | 888 | 1094 | 1369 | 2049 | 2463 | 1434 | 1.26E-02 | 1.78 | Retrotransposon |
| *CIRC* | 18 | 30 | 23 | 51 | 62 | 56 | 3.00E-03 | 2.43 | Retrotransposon |
| *COPIA* | 23126 | 62779 | 58084 | 126439 | 166397 | 83807 | 3.97E-03 | 2.62 | Retrotransposon |
| *CR1A* | 178 | 192 | 223 | 329 | 334 | 207 | 8.83E-02 | 1.47 | Retrotransposon |
| *Diver* | 58 | 39 | 55 | 43 | 42 | 62 | 9.25E-01 | 0.96 | Retrotransposon |
| *DIVER2* | 18 | 32 | 26 | 30 | 26 | 20 | 9.54E-01 | 1.03 | Retrotransposon |
| *DM_ROO* | 510 | 1014 | 1140 | 1350 | 1226 | 769 | 5.00E-01 | 1.26 | Retrotransposon |
| *DM88* | 38 | 54 | 43 | 44 | 42 | 20 | 5.81E-01 | 0.80 | Retrotransposon |
| *DMGYPF1A* | 166 | 141 | 165 | 274 | 300 | 277 | 4.80E-04 | 1.80 | Retrotransposon |
| *DOC* | 2636 | 1966 | 2108 | 3450 | 3628 | 2834 | 4.25E-02 | 1.48 | Retrotransposon |
| *DOC2* | 225 | 167 | 177 | 211 | 185 | 176 | 9.81E-01 | 1.00 | Retrotransposon |
| *DOC3* | 231 | 276 | 326 | 462 | 457 | 333 | 4.49E-02 | 1.51 | Retrotransposon |
| *DOC4* | 14 | 26 | 24 | 30 | 87 | 30 | 5.48E-02 | 2.32 | Retrotransposon |
| *DOC5* | 11 | 26 | 35 | 33 | 25 | 17 | 8.96E-01 | 1.08 | Retrotransposon |
| *F-element* | 385 | 382 | 421 | 529 | 446 | 361 | 5.95E-01 | 1.13 | Retrotransposon |
| *Flea* | 733 | 737 | 825 | 1431 | 1294 | 783 | 6.13E-02 | 1.53 | Retrotransposon |
| *FROGGER* | 25 | 63 | 49 | 49 | 16 | 17 | 3.25E-01 | 0.61 | Retrotransposon |
| *FW2* | 18 | 31 | 29 | 23 | 23 | 10 | 5.36E-01 | 0.74 | Retrotransposon |
| *FW3* | 15 | 30 | 30 | 21 | 13 | 10 | 2.82E-01 | 0.60 | Retrotransposon |
| *G2* | 58 | 39 | 49 | 61 | 71 | 56 | 4.40E-01 | 1.28 | Retrotransposon |
| *G3* | 13 | 17 | 28 | 12 | 7 | 9 | 1.69E-01 | 0.51 | Retrotransposon |
| *G4* | 71 | 145 | 124 | 126 | 74 | 76 | 5.83E-01 | 0.82 | Retrotransposon |
| *G5* | 98 | 73 | 93 | 97 | 88 | 101 | 8.04E-01 | 1.08 | Retrotransposon |
| *G5A* | 138 | 158 | 182 | 169 | 135 | 126 | 7.02E-01 | 0.90 | Retrotransposon |
| *G6* | 24 | 21 | 31 | 37 | 59 | 57 | 2.96E-02 | 2.02 | Retrotransposon |
| *Gate* | 166 | 121 | 166 | 362 | 484 | 472 | 1.00E-08 | 2.90 | Retrotransposon |
| *GTWIN* | 14 | 12 | 16 | 20 | 39 | 16 | 2.10E-01 | 1.78 | Retrotransposon |
| *GYPSY10* | 90 | 135 | 101 | 118 | 84 | 83 | 6.83E-01 | 0.88 | Retrotransposon |
| *GYPSY11* | 10 | 7 | 24 | 12 | 11 | 10 | 1.00E+00 | 0.81 | Retrotransposon |
| *GYPSY2* | 29 | 29 | 21 | 36 | 37 | 42 | 2.88E-01 | 1.46 | Retrotransposon |
| *GYPSY4* | 41 | 50 | 56 | 67 | 82 | 49 | 3.59E-01 | 1.35 | Retrotransposon |
| *GYPSY5* | 4 | 6 | 11 | 16 | 15 | 8 | 1.00E+00 | 1.85 | Retrotransposon |
| *GYPSY6* | 12 | 20 | 25 | 27 | 26 | 17 | 6.08E-01 | 1.28 | Retrotransposon |
| *GYPSY8* | 38 | 25 | 28 | 12 | 25 | 31 | 5.43E-01 | 0.75 | Retrotransposon |
| *GYPSY9* | 37 | 32 | 54 | 42 | 13 | 13 | 2.27E-01 | 0.56 | Retrotransposon |
| *HeT-A* | 1033 | 1077 | 1180 | 1182 | 1283 | 798 | 9.78E-01 | 0.99 | Retrotransposon |
| *I-element* | 224 | 201 | 197 | 418 | 372 | 208 | 5.26E-02 | 1.61 | Retrotransposon |
| *Idefix* | 72 | 136 | 193 | 243 | 352 | 127 | 1.08E-01 | 1.81 | Retrotransposon |
| *INVADER* | 46 | 57 | 42 | 35 | 34 | 29 | 2.34E-01 | 0.68 | Retrotransposon |
| *INVADER2* | 38 | 66 | 77 | 140 | 192 | 118 | 9.10E-04 | 2.50 | Retrotransposon |
| *INVADER3* | 150 | 176 | 185 | 241 | 336 | 196 | 7.21E-02 | 1.51 | Retrotransposon |
| *INVADER4* | 19 | 34 | 37 | 61 | 77 | 47 | 2.58E-02 | 2.07 | Retrotransposon |
| *IVK* | 53 | 55 | 45 | 125 | 225 | 224 | 1.00E-07 | 3.73 | Retrotransposon |
| *Jockey* | 848 | 596 | 653 | 831 | 1122 | 1106 | 6.34E-02 | 1.46 | Retrotransposon |
| *Jockey_1* | 579 | 539 | 644 | 519 | 542 | 409 | 3.68E-01 | 0.83 | Retrotransposon |
| *JOCKEY2* | 99 | 152 | 146 | 98 | 83 | 47 | 5.90E-02 | 0.58 | Retrotransposon |
| *JUAN* | 80 | 96 | 97 | 165 | 265 | 133 | 4.37E-03 | 2.07 | Retrotransposon |
| *Max-element* | 859 | 779 | 364 | 1190 | 807 | 781 | 3.00E-01 | 1.39 | Retrotransposon |
| *MDG3* | 712 | 708 | 553 | 925 | 753 | 408 | 8.72E-01 | 1.06 | Retrotransposon |
| *MGD1* | 178 | 350 | 425 | 553 | 833 | 473 | 1.94E-02 | 1.95 | Retrotransposon |
| *Microcopia* | 35 | 36 | 43 | 44 | 45 | 21 | 9.68E-01 | 0.98 | Retrotransposon |
| *ninja* | 33 | 28 | 39 | 17 | 33 | 23 | 4.44E-01 | 0.73 | Retrotransposon |
| *OPUS* | 1450 | 1511 | 1833 | 2025 | 2093 | 2767 | 6.66E-02 | 1.44 | Retrotransposon |
| *QBERT* | 17 | 30 | 31 | 36 | 42 | 47 | 1.74E-01 | 1.63 | Retrotransposon |
| *QUASIMODO* | 40 | 66 | 63 | 91 | 100 | 42 | 3.90E-01 | 1.39 | Retrotransposon |
| *R1-2* | 19 | 14 | 18 | 15 | 3 | 7 | 1.91E-01 | 0.50 | Retrotransposon |
| *R1A1-element* | 44 | 28 | 30 | 48 | 48 | 55 | 2.53E-01 | 1.46 | Retrotransposon |
| *ROOA_LTR* | 56 | 60 | 88 | 209 | 276 | 150 | 1.00E-05 | 3.13 | Retrotransposon |
| *ROVER* | 55 | 110 | 156 | 146 | 200 | 71 | 5.52E-01 | 1.30 | Retrotransposon |
| *Rt1a* | 27 | 41 | 32 | 55 | 54 | 30 | 3.64E-01 | 1.41 | Retrotransposon |
| *RT1B* | 136 | 171 | 167 | 183 | 212 | 143 | 6.19E-01 | 1.14 | Retrotransposon |
| *RT1C* | 26 | 19 | 25 | 19 | 19 | 20 | 6.90E-01 | 0.83 | Retrotransposon |
| *S-element* | 89 | 150 | 124 | 163 | 202 | 100 | 4.42E-01 | 1.28 | Retrotransposon |
| *S2* | 17 | 20 | 31 | 26 | 51 | 15 | 5.35E-01 | 1.38 | Retrotransposon |
| *SPRINGER* | 1363 | 1611 | 1080 | 2201 | 1600 | 1522 | 2.48E-01 | 1.31 | Retrotransposon |
| *STALKER2* | 55 | 145 | 139 | 115 | 112 | 79 | 8.19E-01 | 0.91 | Retrotransposon |
| *TABOR* | 41 | 126 | 162 | 131 | 106 | 54 | 8.24E-01 | 0.89 | Retrotransposon |
| *TARTC* | 56 | 45 | 72 | 107 | 183 | 94 | 5.13E-03 | 2.22 | Retrotransposon |
| *TOM1_LTR* | 28 | 52 | 76 | 66 | 105 | 69 | 2.58E-01 | 1.53 | Retrotransposon |
| *Transpac* | 458 | 1106 | 1447 | 2514 | 2842 | 820 | 2.94E-01 | 2.05 | Retrotransposon |
| *X-ELEMENT* | 68 | 45 | 55 | 62 | 53 | 55 | 9.78E-01 | 1.01 | Retrotransposon |
| *ZAM* | 29 | 35 | 37 | 27 | 21 | 27 | 4.38E-01 | 0.74 | Retrotransposon |
| *1360* | 416 | 775 | 710 | 1240 | 1224 | 734 | 4.76E-02 | 1.68 | DNA |
| *BARI1* | 27 | 46 | 40 | 70 | 67 | 48 | 1.12E-01 | 1.65 | DNA |
| *Hobo* | 90 | 185 | 138 | 175 | 188 | 155 | 4.45E-01 | 1.26 | DNA |
| *Pogo* | 216 | 500 | 487 | 612 | 774 | 365 | 2.69E-01 | 1.46 | DNA |
| *HB* | 78 | 148 | 151 | 178 | 200 | 157 | 2.08E-01 | 1.42 | DNA |
| *FB* | 98 | 177 | 215 | 152 | 259 | 105 | 9.03E-01 | 1.06 | DNA |
| *Hopper* | 9 | 37 | 20 | 30 | 41 | 23 | 4.70E-01 | 1.45 | DNA |
| *INE1* | 88 | 126 | 133 | 168 | 173 | 109 | 3.43E-01 | 1.31 | DNA |
| *MARINER2* | 84 | 113 | 114 | 98 | 81 | 89 | 6.01E-01 | 0.86 | DNA |
| *P-element* | 339 | 561 | 593 | 739 | 775 | 618 | 1.21E-01 | 1.43 | DNA |
| *TC1* | 89 | 112 | 75 | 144 | 171 | 58 | 4.30E-01 | 1.35 | DNA |
| *TC1-2* | 84 | 111 | 115 | 141 | 166 | 101 | 3.10E-01 | 1.32 | DNA |
| *TRANSIB2* | 35 | 86 | 70 | 61 | 52 | 23 | 4.49E-01 | 0.71 | DNA |
| *TRANSIB3* | 37 | 102 | 77 | 168 | 194 | 92 | 2.99E-02 | 2.12 | DNA |
| *TRANSIB4* | 14 | 14 | 12 | 23 | 17 | 9 | 7.19E-01 | 1.22 | DNA |
